# Assessing the state of obesity care: Quality, access, guidelines, and standards

**DOI:** 10.1002/osp4.765

**Published:** 2024-07-17

**Authors:** Lee M. Kaplan, Caroline M. Apovian, Jamy D. Ard, David B. Allison, Louis J. Aronne, Rachel L. Batterham, Luca Busetto, Dror Dicker, Deborah B. Horn, Aaron S. Kelly, Jeffrey I. Mechanick, Jonathan Q. Purnell, Ximena Ramos‐Salas, for the Assessing the State of Obesity Care Writing Group

**Affiliations:** ^1^ Obesity, Metabolism and Nutrition Institute Massachusetts General Hospital and Harvard Medical School Boston Massachusetts USA; ^2^ Division of Endocrinology, Diabetes, Nutrition and Weight Management Nutrition and Weight Management Center Boston Medical Center and Boston University School of Medicine Boston Massachusetts USA; ^3^ Department of Epidemiology and Prevention Wake Forest University School of Medicine Winston‐Salem North Carolina USA; ^4^ Department of Epidemiology and Biostatistics Indiana University School of Public Health‐Bloomington Bloomington Indiana USA; ^5^ Department of Medicine Weill‐Cornell College of Medicine New York New York USA; ^6^ Department of Medicine Centre for Obesity Research Rayne Institute University College London London UK; ^7^ National Institute for Health and Care Research University College London Hospitals Biomedical Research Centre London UK; ^8^ Department of Medicine University of Padova Padova Italy; ^9^ Department of Internal Medicine D Haharon Hospital Rabin Medical Center Sackler School of Medicine Tel Aviv University Tel Aviv Israel; ^10^ Center for Obesity Medicine and Metabolic Performance University of Texas McGovern Medical School Houston Texas USA; ^11^ Department of Pediatrics and Center for Pediatric Obesity Medicine University of Minnesota Minneapolis Minnesota USA; ^12^ Marie‐Josée and Henry R. Kravis Center for Cardiovascular Health at Mount Sinai Heart New York New York USA; ^13^ Metabolic Support Division of Endocrinology, Diabetes and Bone Disease Icahn School of Medicine at Mount Sinai New York New York USA; ^14^ Division of Endocrinology, Diabetes, and Clinical Nutrition Knight Cardiovascular Institute Oregon Health & Science University Portland Oregon USA; ^15^ Obesity Canada Edmonton Alberta Canada

**Keywords:** obesity care, obesity treatment guidelines, standards of care

## Abstract

**Background:**

An international panel of obesity medicine experts from multiple professional organizations examined patterns of obesity care and current obesity treatment guidelines to identify areas requiring updating in response to emerging science and clinical evidence.

**Aims:**

The panel focused on multiple medical health and societal issues influencing effective treatment of obesity and identified several unmet needs in the definition, assessment, and care of obesity.

**Methods:**

The panel was held in Leesburg, Virginia in September 2019.

**Results:**

The panelists recommended addressing these unmet needs in obesity medicine through research, education, evaluation of delivery and payment of care, and updating clinical practice guidelines (CPG) to better reflect obesity’s pathophysiological basis and heterogeneity, as well as the disease’s health, sociocultural, and economic complications; effects on quality of life; need for standards for quantitative comparison of treatment benefits, risks, and costs; and the need to more effectively integrate obesity treatment guidelines into routine clinical practice and to facilitate more direct clinician participation to improve public understanding of obesity as a disease with a pathophysiological basis. The panel also recommended that professional organizations working to improve the care of people with obesity collaborate via a working group to develop an updated, patient‐focused, comprehensive CPG establishing standards of care, addressing identified needs, and providing for routine, periodic review and updating.

**Conclusions:**

Unmet needs in the definition, assessment and treatment of obesity were identified and a blueprint to address these needs developed via a clinical practice guideline that can be utilized worldwide to respond to the increasing prevalence of obesity.

## 1 INTRODUCTION

Most epidemiologists trace the beginning of the obesity epidemic to the early 1970s with a striking increase in the prevalence and severity of obesity, first in the U.S. and then globally.^1^ Coupled with the recognition of obesity's profound effect on health, longevity, quality of life, and social impacts, these actuarial changes suggested the exacerbation of an extant public health threat. Combined efforts of basic, clinical, and population scientists have subsequently revealed substantial information about obesity's physiological and clinical effects, provided important clues to the current epidemic's causes, yielded more effective treatments, and suggested control strategies.^2^ However, despite multiple public awareness and prevention campaigns, improved dietary and exercise habits, development of safer and more effective medical and surgical obesity therapies, and the establishment and dissemination of numerous clinical practice guidelines (CPG) for prevention and treatment, the prevalence and severity of obesity continue to grow, almost unimpeded.

In September 2019, The Obesity Society (TOS) convened a workshop in Leesburg, Virginia, and invited a panel of 13 recognized leaders in obesity management to review and assess the current state of obesity care and recommend strategies to improve that care. Panelists considered the state of care for people living with obesity by reviewing the efficacy of different obesity therapies, current access to these therapies, disparities in care across populations, barriers to optimal access, and effectiveness of previous efforts to improve care utilization. A major focus was on the organization and content of widely cited CPG^3^, ^4^, ^5^, ^6^ and their contribution to current obesity treatment, particularly potential inconsistencies and gaps in the definition and description of obesity, characterization of obesity‐related risks, strategies for patient‐centered evaluation and treatment outcomes, and criteria for using specific therapies. This report outlines the panel's findings and recommendations for consideration by organizations, clinicians, and public health officials designing CPG and standards of care for people with obesity.

## 2 OBESITY DEFINITIONS, PROGRESSION, AND MEASURES OF SEVERITY

Aware that recognizing obesity as a disease has broad implications for how we address it as a society as well as clinically, panelists reviewed definitions of obesity and epidemiological and clinical markers of its severity; patterns of obesity prevalence and severity in different populations; obesity's clinical, social, and economic implications; and outcomes of previous prevention and treatment strategies. The panel recognized and endorsed the World Health Organization's definition of obesity as a chronic disease characterized by excessive or abnormal adipose tissue that is associated with increased health risk, and reached the consensus that obesity is a disease that develops from a disturbance in the body's normal regulation of fat mass, with development and severity influenced by both endogenous and exogenous (environmental) factors.

Similarly, TOS declared that obesity is a disease on utilitarian grounds. TOS noted that it is not reasonable to empirically show or refute that obesity *is* a disease, but that it is reasonable to conclude that it would be better if obesity were *declared* a disease.^7^ Our committee endorses and accepts TOS's declaration of obesity as a disease. There was more divergence of perspective about whether obesity is a progressive or relapsing disease. While many people with obesity clearly exhibit progressively increased body weight and fat over time, this pattern is not universal. Similarly, whether positive and negative fluctuations in body weight over time reflect changes in obesity's severity (i.e., regression or relapse) or varying use or effectiveness of anti‐obesity therapies remains unclear.

Panelists also recognized that the understanding of obesity as a disease is not universally shared by the public or all professionals working to address obesity. The use of the term *obesity* should only be employed when describing the disease associated with excess or dysfunctional adipose tissue and not when describing body size. Even panel members had varied perspectives: some emphasized the physiological dysregulation promoting storage of excess and/or abnormal body fat, suggesting that any substantial increase in body fat is pathological and therefore a manifestation of disease. Others emphasized the profound medical consequences of obesity, including its association with over 200 other medical conditions, suggesting that excess body fat reflects a disease primarily because of the adverse effects of these complications/comorbidities. The latter view has been facilitated by distinguishing metabolically healthy from unhealthy obesity, with the broader concept “healthy obesity” being used to prevent “medicalizing” obesity and to direct medical efforts instead at complications. Yet, some may consider that there is a logical or linguistic inconsistency in stating that, in some cases, a disease is a healthy condition.

Several panelists also advocated more clearly distinguishing the definition of obesity from the characterization of its severity, arguing that while obesity can be defined merely by the presence of excess body fat, determining severity in a manner that incorporates complications and risks of complications, along with the magnitude of excess body fat and degree of associated dysfunction, would have the benefit of separating metabolically healthy from unhealthy obesity. However, this is not done in other diseases such as diabetes or cardiovascular disease. This is problematic in the case of obesity because of the use of the medical term obesity to describe body size and the disease. The term obesity should be used to describe the disease, and not ever be used to describe body size.

Panelists agreed that current metrics for assessing obesity severity are inadequate for some uses, particularly in the clinical setting. The primary criterion has been the degree of excess body fat, as estimated (but not measured) by body mass index (BMI). A secondary criterion has been body‐fat distribution and adipose function, as estimated (but not measured) by waist circumference or other anthropometric assessments. Ease of measurement and widespread availability in large clinical and epidemiological datasets explain the widespread use of BMI by investigators and public health officials and its adoption by the clinical community as a marker of obesity and a measure of treatment response. Within specific populations, too, BMI is strongly predictive of risk and clinical outcomes.^8^ However, the relationship between BMI and clinical risk varies substantially by population. Even within populations, there is interindividual variability of BMI's predictive power, making it less useful as a measure of obesity severity than it might otherwise be.^9^, ^10^

Panelists therefore recommended identifying and validating additional measures of obesity severity to better predict adverse obesity‐related outcomes, including more direct, imaging‐based measurement of body‐fat mass and distribution, and reflecting the nature and degree of existing medical and psychosocial complications and risk of complications. Integrating these measures into a single severity score to predict overall risk and guide treatment strategies was opined to be more accurate because of the disadvantages of relying on the BMI to assess risk severity. The high clinical value of a single severity or risk score has been demonstrated in numerous other disease states, including most forms of cancer, atherosclerotic cardiovascular disease, heart failure, dyslipidemia, rheumatological disease, alcoholic and non‐alcoholic liver disease, and urgency of the need for solid organ transplantation. The value of this score for obesity lies in its utility in defining modes of treatment based on disease severity.

## 3 ORIGINS OF OBESITY‐RELATED BIAS AND STIGMA

Considering obesity as a disease and understanding its pathophysiological basis are closely related to weight‐ and obesity‐related bias, stigma, and discrimination. The influence appears bidirectional, with misunderstanding of the underlying physiology feeding bias, and bias augmenting structural barriers to effective care (Table 1).^11^ Body weight has long been thought to be controlled by willful behaviors, including food intake and physical activity, leading to a model of body‐weight regulation that balances voluntary energy intake and expenditure. The inference of this model is that obesity largely results from either (1) inappropriate eating and inadequate exercise behaviors, for which the person with obesity is to blame, or (2) changes in the amount and types of food and physical activity accessible in modern environments (e.g., highly processed, highly palatable, calorie‐dense foods promoted by the food industry and/or work/living environments limiting physical activity), which in turn influence personal behavior.

**TABLE 1:** Stigma‐induced barriers to patient care and potential remedies.

Stigma‐induced barriers to patient care	
Provider communication and counseling	Patient care and outcomes
Stigmatizing, insensitive, or blaming language	Patients feel judged and blamed for their weight
Negative weight‐based attitudes and stereotypes	Patients have lower trust in providers
Attributing causes of obesity to personal choices/control	Poorer provider‐patient communication
Weight‐based terminology that patients dislike	Reduced quality of patient care
Lack of patient‐centered communication	Inadequate medical equipment to accommodate patients of diverse body sizes
Inadequate rapport building and lack of empathy	Poorer patient adherence and treatment outcomes
Attributing presenting problems to weight without considering other explanations	Increased clinical attrition
Emphasis on weight or weight loss as only goal	Patient avoidance and delay of care

By reinforcing the perception that longer‐term control of obesity depends on purposeful manipulation of calorie balance, both views overlook decades of evidence that over months to years, body‐fat mass is regulated by physiological processes independent of voluntary eating and exercise behaviors, and that the body's regulation of energy balance powerfully defends its desired fat mass, commonly referred to as the body weight “set point.”^12^, ^13^ Nonetheless, the misperception persists that obesity results from aberrant behavior rather than disordered physiology, blaming the achievement and maintenance of an obese state on the person with obesity. Evidence suggests that behaviors are part of the pathophysiologic mechanisms, and that these behaviors are themselves driven by internal biological signals (e.g., satiety factors and fullness, ghrelin and hunger).

Creating the expectation that both obesity's cause and effective management are under voluntary control exacerbates bias, stigma, and discrimination.^14^, ^15^, ^16^ This misperception also diminishes recognition of obesity's pathophysiological basis, impeding the development and implementation of effective medical treatments and inhibiting people with obesity (many of whom have internalized the ascribed blame) from seeking appropriate care.^17^ Regardless of the pathophysiology of obesity, our panel posits here that discrimination based on size is morally reprehensible whether or not it is recognized as a disease and not a matter of “will power.” We separate here the issue of discrimination based on size as a moral concern and not a matter of understanding the cause of obesity.

This writing group therefore supports the Joint International Consensus Statement on Ending the Stigma of Obesity^15^ that states that obesity stigma is reinforced by lack of awareness of scientific evidence and that obesity stigma is unacceptable in modern societies as it undermines the rights and health of those afflicted due to physical and psychological consequences that allow for less access to care. We posit that academic and professional organizations, the media, public health, and government should encourage education about obesity stigma and facilitate the truthful narrative of obesity based on modern scientific knowledge.

## 4 CLINICAL ASSESSMENT

Limitations and biases associated with the current BMI‐based definition of obesity not only impact clinical practice but also have unintended negative consequences for individuals living with obesity.^18^ Guiding clinical care toward health‐focused targets rather than body size and weight alone requires a more comprehensive definition of obesity, addressing common misconceptions about obesity's etiology and nature and recognizing the primary contribution of obesity to expression and severity of many chronic diseases managed in the clinic, especially prediabetes and type 2 diabetes, thus making it an important target for medical therapy. Although anthropometric measures such as BMI, waist circumference, and waist‐hip ratio can be helpful screening tools, they lack specificity and sensitivity at the individual level and fail to accurately assess relative health risk.^2^ BMI and waist circumference cannot accurately predict comorbidities or functional status. For example, these measurements also fail to reflect evidence that individuals can experience good health at different body sizes. Focusing solely on weight and/or body size may therefore under‐ and over‐diagnose individuals with obesity.

At a cellular level, obesity is the direct result of excess and ectopic accumulation of adipose tissue, a complex heterogenous endocrine organ secreting various physiologically active molecules. For example, abnormal or dysfunctional adiposity can dysregulate metabolic pathways and impair health.^19^ Nonetheless, diagnosing obesity solely on the amount of excess fat—besides the impracticality of measurement in most primary care settings—is equally insufficient because the amount, distribution, and function of an individual's adiposity affect its impact.^3^ Properly diagnosing obesity requires assessing body‐fat distribution as well as determining ways that adipose tissue affects health. For example, central obesity presents a higher‐risk for diseases such as type 2 diabetes, metabolic syndrome, and heart disease, whereas peripheral obesity fat distribution (around the hip and thigh areas) may be more protective—suggesting that diagnosing obesity requires a comprehensive workup and assessment to identify the obesity phenotype.^20^

Equally important is assessing obesity's consequences and severity before determining a treatment path.^4^ Here, too, the anthropomorphic definition falls short. Responses to obesity treatments can vary from person to person both in terms of the amount of weight loss and improvements in obesity‐related complications. Measuring response or outcome on based solely on body weight or size can also overlook important health benefits associated with treating obesity and its associated conditions.^21^ Conceptualizing obesity treatments beyond weight loss/body size toward improving health and patient‐centered goals would be more consistent with chronic disease management principles.^5^ For this reason, classifications of obesity incorporating obesity‐related complications predict individual health risk and mortality better than those using BMI.

Developing those more specific classifications will require low‐cost and readily available imaging methods allowing accurate, BMI‐independent determination of body composition and fat distribution, along with outcome studies utilizing these methods to confirm weight‐ and adiposity‐specific targets of obesity therapies to optimize long‐term health.^20^ Combining genetic, metabolic, lipidomic, and microbiome studies with more advanced imaging methodologies holds potential for further individualization of prognosis and treatment approaches. Until a convenient measure is validated to replace BMI which more accurately reflects the risk associated with excess body fat, this expert panel supports the American Medical Association's recent policy urging health care practitioners to avoid using BMI alone when evaluating patients.^22^

## 5 ASSESSING LONG‐TERM OUTCOMES AND RISKS

Advances in imaging techniques show that adverse health outcomes of excess adiposity are influenced not only by total fat but also by relative amount of fat (e.g., percent body fat), its regional distribution (e.g., truncal vs. peripheral, visceral vs. subcutaneous), and/or ectopic lipid accumulation (e.g., liver, muscle, pancreatic). In addition, advances in the science of the physiology of weight regulation and adipocyte physiology have begun illuminating pathophysiological links between excess adiposity and functional impairment both at the organ and systemic levels.

Variations in body composition and fat distribution are thought to underlie differences in expression of obesity complications and comorbidities among sexes, races and ethnicities across the lifespan.^6^, ^23^, ^24^, ^25^ Although measures of regional body weight, such as waist circumference and waist‐to‐hip ratio, trend with those of generalized adiposity, such as BMI, regional measures have incremental predictive value to BMI for adverse health outcomes.^26^, ^27^ This is especially important for patients, including many of Asian origin, with central adiposity who otherwise fall into a “healthy” weight range using BMI criteria.^28^, ^29^, ^30^ Health risks and complications resulting from excess adiposity can thus occur at any level of BMI.

Obesity's adverse health impact involves every organ.^31^ As a mechanical function of increasing weight, people with obesity can experience long‐term irreversible damage to weight‐bearing joints, reducing functional status in patients with conditions affecting ambulation such as strokes or multiple sclerosis. Patients can also experience gastroesophageal reflux, obstructive sleep apnea, and restrictive lung disease. The most closely linked metabolic complications of obesity include dyslipidemia, prediabetes, type 2 diabetes, and hypertension (HTN), all of which contribute to increased risk for adverse cardiovascular outcomes, including not only cardiovascular disease but also cardiac rhythm issues, epicardial adipose tissue, ventricular dysfunction, and heart failure. Furthermore, while so‐called “metabolically healthy” people with obesity may lack measurable adverse effects on lipid or glucose levels, they remain at an increased cardiovascular risk long‐term.^32^, ^33^ Ectopic liver fat in the form of non‐alcoholic fatty liver disease increases with excess body weight gain. NAFLD—hepatic steatosis on imaging or histology in the absence of known causes—is rapidly becoming the most common cause of chronic liver disease worldwide.^34^ In addition, there is evidence that obesity is associated with cancer incidence.^35^ Therefore “metabolically healthy” persons with obesity should be treated for primary prevention, as opposed to reserving medical and surgical therapies until later in disease processes when patients are dealing with often irreversible obesity complications and residual risk, as is alluded to by guidelines based on the Edmonton classification.^36^

Obesity can also increase the risk of cognitive dysfunction, depression, and anxiety as well as diminish the quality of life.^37^, ^38^, ^39^, ^40^ Endocrine dysfunctions, including hypogonadism, polycystic ovarian syndrome, and infertility, are common in patients with obesity, and low levels of sex steroids and growth hormone may exacerbate body composition abnormalities. Obesity in pregnancy adversely impacts both maternal and fetal health, increasing rates of gestational diabetes, hypertensive disorders of pregnancy, large‐ and small‐for‐gestational‐age babies, and macrosomia, as well as increasing risk for childhood obesity.^36^, ^41^ Finally, obesity worsens both disease‐specific and total mortality rates in both men and women.^42^, ^43^, ^44^

## 6 OUTCOMES OF OBESITY TREATMENTS

A weight loss of as little as 3%–5% improves some obesity‐related diseases, and >10% improves others; however, additional research is still needed to determine how much weight loss is required to engender improvement/remission of different complications for people at different stages of obesity and phases of life. It is already clear that patients can expect some degree of weight loss using any or all the following approaches: lifestyle modification (LM), pharmacotherapy, metabolic and bariatric surgery (MBS), and devices (including endoscopic procedures). Each approach is accompanied by different clinical expectations, not all of which are fully understood by physicians or accurately conveyed to patients.^3^, ^23^

While the efficacy of comprehensive LM programs that encompass a variety of dietary approaches, physical and behavioral counseling, settings, and populations has been established,^45^ these programs are best viewed as part of a broader treatment toolbox and used to maximize treatment response to other therapies. Several factors affect outcomes achieved with LM, including at the patient level, demographics, social determinants of health, medical history, disease severity, use of weight‐promoting medications, and mental health, and at the treatment level, mode, intensity/frequency, interventionist skill, setting, and treatment complexity. Most studies show that the average weight loss achieved by adults through these programs after 6 months is 5%–10% of the initial weight.^46^ In addition, patients must expect weight gain at the rate of 1–2 kg per year following weight loss, partly due to activation of compensatory physiological mechanisms which contribute to regaining weight.^47^, ^48^, ^49^

Ongoing follow‐up with obesity medicine specialists can attenuate weight regain and rapid weight regain, which can lead to cardiovascular detriment due to oxidative stress.^50^ Although this weight regain can be attenuated with ongoing behavioral therapy, clearly communicating the expected weight loss and weight regain to patients may not promote greater success.^51^, ^52^ Doing otherwise, however, would not fulfill the ethical obligation of respect for persons,^53^ which requires honesty and respect for autonomy. In addition, evidence suggests that treating LM as dynamic and adjustable, and including additional therapy from the outset when expected magnitude of response is less than desired leads to better treatment responses.^54^, ^55^ The challenge lies in identifying which people require LM and which type of LM. In addition, the role of technology in delivering LM requires future research. Based on the principle of heterogeneity of obesity causes and impacts, all treatment plans should be tailored to the patient's needs.

Pharmacotherapy is one strategy to offset changes in appetite, satiety, cravings, and energy expenditure and improve adherence to lifestyle interventions. The 2013 American College of Cardiology/American Heart Association/TOS guideline for managing overweight and obesity in adults,^46^ the American Association of Clinical Endocrinologists,^3^ and the Endocrine Society's CPG on the pharmacologic management of obesity^56^ all recommend pharmacotherapy for the treatment of adult obesity if a patient has a BMI ≥ 30 kg/m^2^ or a BMI ≥ 27 kg/m^2^ with weight‐related complications, such as HTN, dyslipidemia, type 2 diabetes, and obstructive sleep apnea. Those who treat obesity no longer view the historical approach of AOMs as “adjunct” therapy to LM as adequate, but that lifestyle and AOMs should be started simultaneously for those who qualify and would most benefit. This is aligned with American Diabetes Association standards of care, which now recommend simultaneously initiating metformin and comprehensive lifestyle management when A1c > 6.5%.^57^ Until 2013, orlistat was the only obesity medication approved by regulatory agencies worldwide, along with phentermine (for short‐term treatment) in the U.S. Newer obesity medications have subsequently been approved for long‐term treatment, making effective and safe obesity medications increasingly available, although access varies regionally. At present, the FDA‐approved obesity medications in the U.S. are phentermine, orlistat, phentermine/topiramate extended‐release, naltrexone sustained release (SR)/bupropion SR, injectable liraglutide 0.60–3.0 mg, semaglutide 0.25–2.4 mg and tirzepatide 2.5–15 mg. Metformin is often used “off‐label” to treat obesity, although the mean treatment effect is small.^58^ Adolescent clinical trials have resulted in the regulatory approval of orlistat and liraglutide,^59^ the combination of phentermine/topiramate extended,^60^ and semaglutide approval for adolescents older than 12 years of age.^61^

Successful pharmacotherapy for obesity depends on tailoring treatment to patients' individual behaviors, socioeconomic and cultural contexts, and complications, as well as closely monitoring efficacy, safety, and tolerability. Weight loss among adults varies with both specific medication and population treated, but on average 35%–65% of those treated with phen/top, bup/nalt, and liraglutide medications can expect 10% average weight loss or more after 1 year.^57^ The second‐generation medications, beginning an era of >10% weight loss with semaglutide and the type 2 diabetes medication tirzepatide, are coming closer to bariatric surgery average weight losses with 16%–17% and 19%–20% respectively.^58^, ^59^

Because obesity is a chronic and often progressive condition, patients should be given the expectation from the beginning that these medications will be continued lifelong and that a weight plateau represents establishment of therapeutic efficacy for that mediation, rather than drug failure. Because each type of medication acts via a different mechanism, substituting an alternative medication or adding a second medication if a healthy weight is not achieved with one will plausibly lead to greater efficacy than adding another medication with the same or similar mechanism as the first medication, in the event that attempts at greater efficacy are judged to be merited if the desired treatment response is not achieved. Such combination medical therapy is now the norm for other chronic diseases such as type 2 diabetes and HTN.^58^, ^60^

Interventional trials of lifestyle or pharmacologic management for overweight or obesity have failed to demonstrate a reduction in cardiovascular events until the Semaglutide Effects on Cardiovascular Outcomes in People with Overweight or Obesity (SELECT) trial resulted in a 20% reduction in major adverse cardiac events, composed of CV death, nonfatal myocardial infarction, and stroke (MACE), compared with placebo.^60^ The SELECT trial solidifies that the use of semaglutide in those with overweight or obesity should take its place alongside other standard evidence‐based practices such as pharmacologic treatment of HTN, diabetes, and dyslipidemia for secondary atherosclerotic cardiovascular disease prevention.^62^ We await cardiovascular outcomes for other second‐generation therapies ongoing and in the future.

Metabolic and bariatric surgery has emerged as a durable obesity therapy currently in use for extreme BMI categories. These surgeries, the first highly effective obesity treatments since their inception in the 1950s, can produce meaningful and sustained weight loss with simultaneous improvement or remission in many obesity‐related complications. Indeed, the most convincing evidence regarding the benefits of obesity management on morbidity and mortality comes from studies of surgical intervention, which alters gut‐derived signals regulating energy and glucose homeostasis. Long‐term average weight loss in adults following the most common current metabolic/bariatric procedures ranges between 20% and 30%,^63^ exceeding long‐term average weight loss achieved through lifestyle (∼4%) and pharmacologic treatment (6%–15%). On average, Roux‐en‐Y Gastric Bypass results in 25%–30% weight loss in the long term, and sleeve gastrectomy (SG) 20%–25%.^64^

These surgeries produce subsequent health benefits for nearly every organ system, especially disease remission or reductions in drug treatment burden for type 2 diabetes, dyslipidemia, HTN, obstructive sleep apnea, atherosclerotic disease, and heart failure,^65^ with reduced rates of several cancers reportedly associated with weight loss following MBS.^66^ Recent studies, including the prospective Teen‐Longitudinal Assessment of Bariatric Surgery Study and the Adolescent Morbid Obesity Surgery Study, show that MBS is effective in adolescents with obesity as well, producing similar marked weight reduction and health improvements as in adults.^67^, ^68^ Other evidence shows weight‐loss‐independent effects of MBS and new pharmacotherapies that warrant further research.^64^, ^69^

Metabolic and bariatric surgery is also associated with marked improvement in obesity's metabolic and mechanical complications as well as improved quality of life, with observational cohort studies showing marked reductions in cardiovascular and all‐cause mortality.^65^, ^70^, ^71^, ^72^, ^73^ Over 10 randomized controlled trials (RCTs) to date comparing best medical management to MBS in patients with obesity and type 2 diabetes markedly favor MBS in terms of glycemic control and medication usage,^74^ with long‐term reductions in micro‐ and macrovascular complications^75^ in agreement with observational studies.^76^ Consequently, MBS is now recognized as part of the management algorithm for type 2 diabetes in the United Kingdom's National Institute for Health and Care Excellence guidelines advocating for surgery early in the course of diabetes progression.^77^

Historically, adult patients were considered eligible for metabolic/bariatric surgery starting at a BMI ≥ 40 kg/m^2^ or >35 kg/m^2^ with an obesity‐related complication. Updated American Society of Metabolic and Bariatric Surgery guidelines recommend MBS for those with BMI > 35 kg/m^2^ regardless of the presence, absence, or severity of comorbidities. MBS should be considered for those with metabolic disease and a BMI of 30–34.9 kg/m^2^.^78^ For these populations, MBS is now recognized as the most effective treatment. However, health benefits of these procedures have been demonstrated down to BMIs of 30 kg/m^2^, and possibly even lower values.^74^, ^78^

For example, in the Asian population, the prevalence of diabetes and cardiovascular disease is higher at a lower BMI than in the non‐Asian population. Thus, in Asians, BMI risk should be adjusted to define obesity at a BMI of 25–27.5 kg/m^2^. In certain populations, access to MBS should not be denied solely based on the traditional BMI thresholds.^79^

Large cohort studies have accordingly shown reduced disease‐specific and total mortality rates in patients who have undergone MBS, often exceeding rates published for best current medical therapies of these diseases.^65^, ^80^ Limitations of these approaches include variable treatment responses across different obesity phenotypes, the need for long‐term nutrient supplementation, and potential adverse mental‐health effects (depression, suicidality, and substance abuse in the case of specific procedures). With widespread application of laparoscopic techniques and improved surgical training and practice standards in recent years, operative mortality and complications are low, but lifelong follow‐up is needed. Given obesity's progressive nature, moreover, weight gain with time is expected, and patients may require additional therapies such as obesity medications. Although improved safety and outcomes have made surgery a far more attractive option for long‐term management of severe and complicated obesity, misunderstandings that MBS promotes weight loss by limiting ingestion or absorption of macronutrients, which are held despite early demonstrations that surgery worked primarily by improving endogenous physiology,^81^ have limited wider acceptance by reinforcing perceptions of patient responsibility and exacerbating weight bias and stigma.

A fourth treatment approach to obesity that remains less explored involves various devices and endoscopic procedures currently in development or clinical trials. These include space‐occupying devices such as gastric balloons, endoscopic gastroplasty, aspiration technology, post‐metabolic and bariatric surgery endoscopic revision, and obesity‐related natural orifice transluminal endoscopic surgery procedures.^82^ Future research is needed to establish the place for these in the toolbox of obesity management strategies, especially in light of positive safety and efficacy results for the endoscopic SG in over 200 subjects in the MERIT trial.^83^

## 7 ASSESSING CPG

The expanding role of psychological therapies, pharmacotherapy, and surgical procedures in treating obesity is consistent with the clinical reframing of obesity as a chronic disease. However, this reframing remains challenged not only by problems with definitions and incomplete understanding of the interaction between the physiological, environmental, and psychosocial factors involved in the etiology and progression of the disease but also by a lack of consistent and comprehensive CPG with prescriptive recommendations for clinical care. Currently the various CPGs produced by many different professional medical organizations outline treatment by addressing issues including chronic‐care models of adiposity‐based disease; screening, case finding, and risk stratification; roles for early and sustainable prevention; physiological mechanisms; social, environmental, and psychosocial factors; the roles of psychological therapy, behavioral interventions (e.g., medical nutrition therapy, physical activity interventions, stress management interventions), and behavior change interventions (strategies to support changes in behaviors that support obesity management); pharmacotherapy; surgical procedures; and hard and realizable endpoints. However, each of the various CPGs targets different aspects of obesity, based on different terms, definitions, and methodologies and resulting in inconsistent conclusions as well as lack of compelling validation to optimize clinical practice. In contrast to guidelines for other chronic diseases (e.g., HTN or diabetes), obesity CPGs tend to include recommendations that are permissive (i.e., state what clinicians might consider recommending) rather than prescriptive (i.e., recommending a specific course of action).

Current treatment guidelines seem less directive for treatment when patients with obesity are seen in a primary care provider's office as compared to guidelines for other diseases. Figure 5 of the 2016 AACE/ACE Obesity CPG,^3^ for example, uses the verbs “consider” and “suggested” to guide therapy based on BMI and stage of complications, in contrast to the 2017 ACC/AHA HTN guidelines, which use the verbs “identify,” “discontinue,” “screen,” “maximize,” and “add” for treatment resistance and medication prescribing and the term “refer” regarding secondary causes of HTN.^84^ Contributing to the lack of clear treatment‐strategy directives is the inability of the stratification tool for patients with obesity, the BMI, to determine true risk. The lack of prescriptive treatment imperatives creates ambiguity that may allow third‐party payors greater latitude in determining coverage, which is often lacking or inconsistent with available evidence. These coverage gaps create disparities for patients unable to afford therapy and reinforce inaction by the healthcare practitioner, who is incentivized to focus on health concerns that have defined metrics and outcome targets to assess performance.

Across all existing CPGs for treating obesity, the evidence base is largest for behavior‐based therapy; however, the dose (frequency of contact) demonstrated to have even modest efficacy for adults by the United States Preventive Services Task Force^29^ may be impractical from both patient and payor perspectives: clinical outcomes of tertiary‐care weight‐management programs are small in terms of weight loss (averaging 4%–8% of total body weight) and labor intensive for healthcare providers.^30^ However, it is now understood that second‐generation therapies treat obesity by reducing energy intake by markedly enhancing satiation and decreasing hunger, and these therapies appear to lessen the need for traditional cognitive and behavioral strategies (e.g., monitoring food intake) to achieve calorie restriction.^85^

Current CPGs have embraced MBS for treating patients with obesity given its established benefits to health outcomes and mortality, recommending consideration as first‐line therapy for patients with a BMI ≥ 50 kg/m^2^ as well as with type 2 diabetes and BMI > 35 kg/m^2^ because of the specific glucometabolic benefits from these procedures and otherwise high rate of obesity‐related complications in non‐surgical control groups.^71^, ^77^, ^79^, ^86^ Nonetheless, uptake overall entails only 1%–2% of medically eligible U.S. patients and 1.0% of adults and <0.1% of adolescents globally.^81^, ^83^, ^87^, ^88^ The results of the SELECT trial showing a reduction in cardiovascular events with semaglutide treatment in overweight/obesity without diabetes demands the rewriting of obesity CPGs in a prescriptive manner to reflect decreased mortality with pharmacotherapy for obesity.

Various organizations have published pediatric obesity CPGs, including the American Academy of Pediatrics,^89^ and the Expert Committee recommendations authored by representatives from 15 different societies,^90^ including the Endocrine Society,^91^ the American Society for MBS,^92^ and Obesity Canada.^93^ Unlike adult CPG recommendations, treatment goals for pediatric obesity remain unclear, partly owing to the wide array of different BMI outcomes used in clinical trials. Although MBS (gastric bypass, and gastric sleeve) is objectively the most effective treatment in adolescents as in adults, current guidelines reserve it for those with the most severe forms of obesity.^94^, ^95^ Finally, the rapid expansion and availability of new anti‐obesity medications for adolescents will necessitate updates to pediatric CPGs in the years to come.

Attempts to develop future guidelines to address many of these gaps might begin with the premise that its recommendations should (1) reflect objective and thorough evaluations of pertinent scientific evidence; and (2) clearly communicate the state of that evidence and the degree to which it suggests or demonstrates the truth of pertinent propositions. Such a premise does not mean that every recommendation necessarily rests on an evidence base *demonstrating* the truth of every pertinent proposition. While holding supporting evidence to a high standard of scientific certainty is highly desirable, it is not essential. As with many if not most other clinical and public health guidelines, in fact, recommendations for the clinical care of obesity might be based on standards of evidence ranging anywhere from informed intuition (i.e., clinical judgment) to the highest standards of “scientific warrant achievable”^96^ such as multiple high‐quality RCTs consistently and unequivocally showing relationships between interventions and outcomes, no contradictory evidence in the literature, and a body of supporting basic science, model organism, mechanistic, and observational studies. What should be necessary for inclusion, however, is clearly communicating the supporting evidence, whatever its state. Again, this is consistent with ethical norms of honesty, respect for persons, and respect for autonomy.

## 8 BARRIERS TO OBESITY CARE—ATTITUDES, BELIEFS, AND KNOWLEDGE GAPS

Obesity remains underdiagnosed and largely undertreated as a disease. At least 27% of women and 31% of men with obesity in the 2003–2008 NHANES survey, for example, were undiagnosed.^97^ Despite the fact that most patients with obesity and healthcare providers agreed that obesity is a disease, only 54%–71% of patients reported discussing weight management with their physician in the last 5 years, and only 21%–24% received weight‐related follow‐up appointments.^17^, ^98^

These low rates of diagnosis, discussion, and treatment reflect numerous barriers to clinical care, including widespread failure to recognize obesity as a chronic disease linked to a failure of physiological mechanisms. Most patients and healthcare providers still believe that lack of exercise and poor food choices are the most relevant barriers to obesity management.^98^ A large international survey by Caterson et al. found that 81% of patients with obesity surveyed believe that weight loss is completely their responsibility, and 71% of 2785 healthcare providers surveyed attribute failure to lose weight to patients' lack of motivation. This survey reported a large time gap (mean 6 years) between the moment when patients started to gain weight and the first discussion about obesity with healthcare providers. Major reasons for this gap were patients' beliefs that managing weight is their personal responsibility and provider perceptions that patients are not interested in, or motivated to, manage their obesity. The notion that physiological forces lead to obesity and weight gain was largely underestimated.^98^

An additional barrier to effective obesity care is the lack of knowledge about effective and available treatments. Most patients and healthcare providers surveyed by Caterson et al. overestimated the long‐term effectiveness of lifestyle modifications (general improvement in eating habits, reducing calories, increasing physical activity) and underestimated the long‐term effectiveness of obesity medication and MBS.^98^ Related to the perception of obesity as a problem linked to personal (lifestyle) choices rather than a physiologically based chronic disease, these findings are reinforced by the emphasis on behavioral modifications in existing CPGs.

Additional barriers to clinical care for obesity include lack of availability, visibility, and accessibility. Most available health resources are directed to prevention rather than effective treatment. Obesity multidisciplinary teams remain rare or understaffed, comprehensive behavioral modification programs and obesity drugs are rarely reimbursed, and access to MBS and post‐surgical follow‐up is limited. In addition, primary care physicians, nurse practitioners, and physician assistants lack the necessary training in obesity medicine to adequately start treatment and follow up patients with obesity. Training in obesity medicine should start in the medical schools and continue into residency and subspecialty fellowships.

## 9 RECOMMENDATIONS AND FUTURE DIRECTIONS

Based on this assessment, the workshop panel reached consensus about steps to enhance equitable access to effective obesity care, including developing:

1. A *definition* of the disease of obesity reflecting excess and/or aberrant adiposity, a physiological basis, and clinical implications (Target Organ Damage).
2. An obesity *severity measure* reflecting the degree of adverse clinical risks and outcomes.
3. Obesity treatment *guidelines* that are prescriptive and provide direct guidance on (a) who should be offered treatment, (b) interventions appropriate to disease severity, (c) treatment goals, and (d) standards of care.
4. Recommendations to promote effective and widespread *implementation* of any newly developed disease definition, severity measures, and treatment guidelines.
5. Recommendations to *reduce barriers* to effective obesity treatment and promote equitable access to treatment.
6. Recommendations to *conduct/implement research* addressing gaps in disease and treatment outcomes, including lack of formal and ongoing education for healthcare providers, and further defining and strengthening obesity treatment guidelines.
7. A mechanism for regularly *updating* obesity treatment guidelines.

As these recommendations suggest, overcoming barriers to effective obesity care will require a systemic multifactorial perspective focused on eliminating the bias and stigma underlying many discriminatory practice patterns. Defining (declaring) obesity as a chronic disease and a healthcare priority by national and international health authorities is a part of this shift. We must overhaul the way society conceptualizes obesity, recognizing it as a chronic, progressive disease, to develop data‐driven, prescriptive treatment guidelines targeted to objective and patient‐oriented health outcomes. In such guidelines, it seems wise to include a clinically useful definition of obesity reflecting an understanding of obesity beyond body size, directing providers to assess obesity's severity and health impact, and reflecting current scientific understanding of the mechanisms underlying obesity's development and maintenance, which likely include homeostatic regulation to some extent.^99^ Including the conception of obesity as a disease with health impacts linked to the interplay among physiological influences, social determinants (which encompass sociocultural practices and beliefs, environmental influences, and public policy), and interindividual‐varying psychological factors may have benefits including reducing stigma.^100^ By displacing blame from the patient for this disease, this reconceptualization removes expectations that patients must earn the right to receive appropriate treatment based on disease severity and facilitates more equitable care by distancing access from personal motivation and resources.

Every segment of the healthcare delivery system from federal policymakers, insurers, health systems, healthcare providers, and the medical industrial complex has a role in shifting blame for obesity and supporting effective care informed by the most rigorous evidence available, and in some cases knowledge of the disease's etiology. Drug‐induced obesity due to iatrogenic effects of pharmacotherapy, for example, might be reduced with alternative labeling of offending medications, additional support for healthcare providers prescribing weight‐neutral alternatives, and broad insurance coverage of treatment options counteracting weight gain, potentially spurring the pharmaceutical industry to produce medications less likely to promote clinically important weight gain. In pediatric populations, comparative effectiveness trials of treatment options that can be scaled up and reach rural patients—for example, telemedicine, mobile apps, and lower‐contact behavioral counseling paired with pharmacotherapy—could help reduce barriers to care as well.

We must also start building healthcare models to trigger routine screening, diagnosis and evaluation, treatment, and long‐term follow‐up for obesity. Doing so will require expanding delivery systems to include comprehensive chronic‐disease‐care models for obesity, including standardizing care pathways and identifying more efficient and cost‐effective care options. Implementing treatment or referral mechanisms for long‐term care wherever individuals with obesity enter the healthcare system (specialty, primary, emergent, or diagnostic/procedural care)—the same model now used for other chronic diseases—is expected to lead to better clinical outcomes.^101^ Because obesity care must focus on long‐term health, moreover, the field needs additional research regarding costs of optimal care for both individuals and populations affected by obesity for all treatment modalities and intensities globally.

We must also shift to an environment where treatment for obesity is regarded as optional. This mindset stems from medical and professional education largely void of opportunities to understand obesity's physiology and chronicity, prevention opportunities, treatment options, and expected outcomes.^102^ Better education about obesity as a chronic physiologically based disease from medical school as well as all healthcare professional programs to continuous medical education could further reduce barriers to care, as could more efforts to communicate the importance of early discussion and intervention in preventing adiposity‐based complications to patients, healthcare providers, and other stakeholders.^103^

Educational standards around medical competency in obesity care—including proficiency testing and continuing medical education requirements—will likely prove valuable globally. Creating a care‐delivery team more proficient in treating obesity requires training in behavioral medicine for all health professionals, including primary care, obstetrics and gynecology, and cardiology. A multidimensional set of pathways to educate early learners in all fields involved in obesity care and in all steps of education, including advanced training in the form of fellowships, will be required, along with equivalent training for other care professionals in multiple forms and levels from basic to advanced.

Efforts to increase knowledge of effective treatment approaches to all relevant stakeholders who work with patients with obesity, to set standards of obesity care by implementing specialized obesity centers,^104^ and to recognize the obesity medicine specialty by the American Board of Medical Specialties and similar bodies are also recommended. If healthcare professionals are familiar with basic principles regarding obesity pharmacotherapy, for example, with providers educated to offer appropriate treatment for a patient's stage of obesity and comorbidities/complications, recognizing that clinically meaningful responses may require switching or adding treatments, the benefits are expected to include improved mortality and quality of life. Efforts should recognize novel second‐generation pharmacotherapies and currently underutilized surgical approaches that alter physiology and accept that reaching treatment goals might not require reaching “normal BMI,” enabling healthcare providers to be clear and honest regarding expected weight loss from different treatment strategies. Improved education about the safety and benefits of MBS—including efforts to correct the message that it “forces people to be good” or is somehow “cheating”—may ameliorate its underutilization.

To achieve these goals, our panel recommends establishing a multidisciplinary, multi‐society working group to develop a new set of comprehensive guidelines for the clinical management of obesity that include the most acute needs for novel research and enhanced professional and public education. Since new information is continuously being published, clinical guidelines, as with all such practice recommendations, are by necessity a work in progress, and must be coupled with a mechanism for regular review, revision, and renewal. They must help guide the healthcare community toward understanding why obesity is a chronic disease that should be addressed similarly to all other chronic, noncommunicable diseases, reflecting the best available evidence to address and reverse its etiology and pathogenesis. Meanwhile, we must continue searching for better ways to assess health and body composition, quantify the efficacy of treatments, and address obesity not as a personal problem but as a complex disease state with systemic contributors. Formal education, including weight bias and sensitivity training, can plausibly correct overt and subconscious barriers to care and improve outcomes. Together, these efforts can help destigmatize obesity treatments and erode the ingrained and deleterious misconception that obesity care rests on individual will.

Indeed, the goals enumerated here were set in motion by the unanticipated popularity and media attention to semaglutide and tirzepatide because of the unprecedented weight losses seen with these agents as well as the long‐term mortality benefit for semaglutide. It seems that the attention to the mechanism of action of these agents has increased the understanding of the community and healthcare providers that obesity is a disease, that these newer AOMs are approaching the efficacy of metabolic surgery, and that metabolic surgery alters hormonal signals in the gut and brain. These are positive events in the understanding of obesity and the need for treatment.

## CONFLICT OF INTEREST STATEMENT

The authors declare no conflicts of interest.

## FINANCIAL DISCLOSURES AT TIME OF MEETING

Dr. Allison has received personal payments or promises for the same from Biofortis, Fish & Richardson, P.C., HawkPartners, IKEA, Laura and John Arnold Foundation, Law Offices of Ronald Marron, Sage Publishing, Tomasik, Kotin & Kasserman LLC, Nestec (Nestlé), and WW (formerly Weight Watchers International LLC). Donations to a foundation have been made on his behalf by the Northarvest Bean Growers Association and the United Soy Bean Board. Dr. Allison is an unpaid member of the International Life Sciences Institute North America Board of Trustees. Dr. Allison's institution, Indiana University, has received funds to support his research or educational activities from: Alliance for Potato Research and Education, Dairy Management Inc., Herbalife, and the NIH. His prior institution, the University of Alabama at Birmingham, received grants, gifts, or contracts from multiple food, beverage, and other for profit and non‐profit organizations with interests in obesity, statistical methods, and research design.

Dr Apovian reported receiving grants from Novo Nordisk; serving on the advisory boards of Orexigen Therapeutics, Gelesis, Allergan, Abbott Nutrition, EnteroMedics, Zafgen, Real Appeal, Nutrisystem, Novo Nordisk, Scientific Intake, Xeno Biosciences, Rhythm Pharmaceuticals, Janssen Pharmaceuticals, Tivity Health, Roman Health Ventures, and Jazz Pharmaceuticals; serving as medical director for Bariatrix Nutrition and SetPoint Health; and serving as a clinical research consultant for Curavit outside the submitted work.

Dr. Ard has received grants or contracts from Nestle Healthcare Nutrition. He has consulted Nestle Healthcare Nutrition, Novo Nordisk, and Amgen. Dr. Ard has received equipment, materials, drugs, medical writing, gifts, or other services from Nestle Healthcare Nutrition.

Dr. Aronne has consulted Amgen, Allurion, Aspire Bariatrics, Eisai, Janssen, Novo Nordisk, Pfizer, and Zafgen Inc. He has an ownership interest in BMIQ, Myos Corporation, Zafgen, Inc., Gelesis, and ERX and serves on the board of directors of Myos Corporation and Jamieson Laboratories.

Dr. Batterham has consulted Novo Nordisk, ViiV, Pfizer and Boehringer‐Ingelheim and participated in clinical trials run by Novo Nordisk.

Dr. Busetto has served on the Advisory Board of Novo Nordisk.

Dr. Dicker has consulted and served on advisory boards for AstraZeneca, Boehringer Ingelheim, Eli Lilly, and Novo Nordisk. He has had research funded to Rabin Medical Center by Eli Lilly, Novo Nordisk, and Rhythm.

Dr. Horn has consulted Eli Lilly, Gelesis, and Novo Nordisk. She has received research funding from Eli Lilly and Novo Nordisk from the University of Texas McGovern Medical School.

Dr. Kaplan has consulted Boehringer Ingelheim, Gelesis, GI Dynamics, Johnson & Johnson, Novo Nordisk, Pfizer and Rhythm.

Dr. Kelly engages in unpaid consulting and educational activities for Boehringer Ingelheim, Eli Lilly, Novo Nordisk, and Vivus. She received donated drugs and placebos from Novo Nordisk and Vivus for NIH‐funded clinical trials.

Dr. Mechanick has received lecture fees from Abbott Nutrition and serves on the advisory boards of Abbott Nutrition, Aveta.Life, and Twin Health. Dr. Purnell has served on advisory boards for Luciole Pharmaceuticals and Novo Nordisk. He also received research funding from the NIH.

Dr. Ramos‐Salas has received grants from the Canadian Institutes of Health Research, Novo Nordisk, and the Social Sciences and Humanities Research Council and received consulting fees from the European Association for the Study of Obesity, Milieu Consulting SRL, Obesity Canada, and World Health Organization. She has received honoraria for lectures, presentations, speaker bureaus, manuscript writing, or educational events from Baricol Bariatrics and Novo Nordisk. Dr. Ramos‐Salas has received support for attending meetings and/or travel from the Canadian Cardiovascular Society, European Association for the Study of Obesity, Novo Nordisk the Obesity Society, and Sociedad Mexicana de Nutricion y Endocrinologia. She is the unpaid chair for Bias180, the unpaid co‐chair for World Obesity Federation Working Group on Weight Stigma, and the unpaid co‐chair for Obesity Canada, Policy Action Team. Dr. Ramos‐Salas is the owner of K&X Ramos AB, a research consulting agency, and is a partner at Replica Communications, a Research and Communications Agency.
